# Impeding factors of early rehabilitation postoperatively after rheumatoid toe arthroplasty: a single-center retrospective cohort study

**DOI:** 10.1186/s40981-020-00356-1

**Published:** 2020-07-07

**Authors:** Shunsaku Goto, Yusuke Kasuya, Keiko Okuyama, Katsunori Ikari, Makoto Ozaki

**Affiliations:** 1 Department of Anesthesiology, Tokyo Women’s Medical University, 8-1 Kawada-cho, Shinjuku-ku, Tokyo, Japan; 2 Department of Anesthesiology, TMG Asaka Medical Center, 1340-1 Mizonuma, Asaka-shi, Saitama, Japan; 3 Department of Anesthesiology, Mejiro hospital, 3-22-23 shimoochiai, Shinjuku-ku, Tokyo, Japan; 4 Department of Orthopedic Surgery, Tokyo Women’s Medical University, 8-1 Kawada-cho, Shinjuku-ku, Tokyo, Japan

**Keywords:** Rheumatoid arthritis, Rehabilitation, Postoperative pain, Peripheral nerve block, Pain management, Walking rehabilitation, Arthroplasty

## Abstract

**Introduction:**

Previous studies explored the benefits related to early ambulation postoperatively, but few focused on patients with rheumatoid arthritis (RA). We retrospectively evaluated the incidence and predictors of the inability to begin walking on the first postoperative day (POD) after toe arthroplasty for rheumatoid arthritis.

**Methods:**

RA patients who underwent toe arthroplasty at one hospital were retrospectively reviewed. A total of 300 patients were included and divided into two groups: possible group (*n* = 191), who were able to walk on the first POD, and impossible group (*n* = 109), who were unable to walk on the first POD. Data were analyzed using odds ratios (OR) with 95% confidence intervals (CI) between various patient factors and the impossible group with logistic regression analysis.

**Results:**

The incidence of postoperative nausea and vomiting before rehabilitation was significantly associated with the infeasibility of walking rehabilitation on the first POD [OR = 2.43, 95% CI 1.22–4.14, *P* = 0.003]. The number of rescue analgesics administered before rehabilitation and the supplementation of peripheral nerve block was also associated with the infeasibility of walking rehabilitation on the first POD [OR = 1.29, 95% CI 1.04–1.59, *P* = 0.003; OR = 0.41, 95% CI 0.20–0.79, *P* = 0.010, respectively].

**Conclusion:**

The incidence of postoperative nausea and vomiting and inadequate postoperative pain management hindered early rehabilitation. Adding peripheral nerve block to general anesthesia had an advantage for postoperative rehabilitation after toe arthroplasty for rheumatoid arthritis.

## Background

Early rehabilitation is beneficial in patients with rheumatoid arthritis (RA) as it shortens hospital stays [1] and reduces infection [2]. Rehabilitation is affected by several factors such as postoperative pain, postoperative nausea and vomiting (PONV), anesthesia methods, types of surgery, and disease severity. In toe arthroplasty for rheumatoid, there are several anesthesia methods. Regional anesthesia for major knee surgery has shown better improvements than general anesthesia (GA) in postoperative rehabilitation [3, 4]. However, these studies were carried out in non-rheumatoid arthritis populations.

In terms of postoperative outcomes, RA patients have different characteristics compared to non-RA patients. Specifically, RA patients are more likely to have worse preoperative and postoperative pain [5] and longer hospital stays after surgery (1). In addition, the majority of RA patients are younger women with a higher risk of PONV.

We hypothesized that postoperative management and anesthesia methods would affect postoperative rehabilitation in RA patients. The purpose of the present study was to investigate predictors of the inability to walk during rehabilitation after rheumatoid toe arthroplasty.

## Methods

The present study was approved by the Institutional Review Board of Tokyo Women’s Medical University (approval number 4416, July 11, 2017). The need for written informed consent was waived due to the retrospective nature of the study. We retrospectively reviewed medical records of all patients with RA who underwent toe arthroplasty at our institute between January 2014 and May 2018.

The inclusion criteria were patients aged 20 years or older, with prior diagnosis of RA, who underwent elective toe arthroplasty, and who were scheduled for their first postoperative day (POD) of rehabilitation. We categorized the patients into two groups: (i) possible group, including those who were able to walk during rehabilitation on the first POD, and (ii) impossible group, including those who were unable to walk during rehabilitation on the first POD.

The primary outcomes were odds ratios (OR) with 95% confidence intervals (CI) between various patient factors and the infeasibility of walking rehabilitation on the first POD. The secondary outcome measures were time until the first rescue analgesic requirement after surgery and an incidence of PONV after each anesthesia method. The post hoc analysis for association between anesthesia methods and postoperative outcome was planned in advance.

The patients’ demographic data including age, sex, height, body weight, preoperative medication for RA, and outcomes (i.e., the information of rehabilitation, postoperative analgesic, PONV, and days from surgery to discharge) were collected by reviewing the patients’ medical records. Intraoperative data such as duration of anesthesia and surgery, anesthesia methods, blood loss and fluid infusion, and total amount of intraoperative fentanyl were collected from the electronic anesthesia record system, Mirrel (FUKUDA DENSHI, Tokyo).

There was a wide variety of anesthesia modalities: GA or non-GA, type of regional anesthesia (i.e., spinal, epidural, or combined spinal-epidural anesthesia), supplementation of peripheral nerve block (PNB), and continuous postoperative opioids.

GA was managed using remifentanil, fentanyl, propofol, sevoflurane, and desflurane. The type and dose of the analgesic were determined by the anesthesiologists in charge. The continuous postoperative intravenous patient controlled analgesia (IV-PCA) was managed by fentanyl (base 15 or 20 μg/h, bolus of 15 or 20 μg, and lockout interval of 10 min). Epidural catheter was placed at the level of L2-L3, L3-L4, L4-L5, or L5/S1 intervertebral space. Patient-controlled epidural analgesia consisted of 0.25% levobupivacaine (base dose of 4 ml/h, bolus of 4 ml, and 30-min lockout interval). The method of single-injection PNB was mainly a combined popliteal sciatic nerve and femoral or subsartorial saphenous nerve block with 20–40 mL of 0.375–0.5% ropivacaine or 0.25% levobupivacaine using ultrasound-guided technique. Two patients were managed by ankle block with 30 ml of 0.5% ropivacaine using ultrasound-guided technique. The combined spinal-epidural anesthesia was administered epidurally at the L2-L3, L3-L4, or L4-L5 intervertebral space and spinal anesthesia at the L3-L4 or the L4-L5 intervertebral space with 2.2–4 ml of 0.5% hyperbaric or plain bupivacaine. The spinal anesthesia was administered at the L3-L4 or the L4-L5 intervertebral space with 2.2–4 ml of 0.5% hyperbaric or plain bupivacaine.

The physical therapist evaluated whether the patient could walk by looking at the patient’s overall condition during rehabilitation on the first POD. Postoperative pain was managed by uniform clinical pathway. There was no regular administration of analgesics, and additional analgesics were administered by the nurse upon the patient request. IVPCA with fentanyl and patient-controlled epidural analgesia bolus was not counted as the number of rescue analgesia.

### Statistical analysis

Data are presented as means (standard deviation), medians (interquartile range), or frequencies (%). Categorical variables were compared using the chi-squared test, and numerical variables were compared using Student’s *t* test for the parametric data and Mann–Whitney *U* test for the non-parametric data.

The primary outcomes were assessed using logistic regression analysis, and the *p* value was calculated with the Benjamini–Hochberg method. We considered that patient factors such as physique, age, sex, daily use of corticosteroid, number of biological disease modifying anti-rheumatic drugs (DMARDs), surgical factors (duration of surgery and blood loss), anesthesia factors (duration of anesthesia, intraoperative fluid volume, anesthesia methods, and intraoperative fentanyl dose), and postoperative factors (postoperative continuous opioid use, postoperative pain, and PONV) hindered postoperative rehabilitation, and comprehensively assessed them using logistic regression analysis.

Time to first rescue analgesic requirement after surgery was compared using the log-rank test and the Kaplan–Meier curve. The incidence of PONV was compared by analysis of variance. Data were analyzed using JMP® Pro 15.0.0 (SAS, Cary, NC, USA). A *p* value < 0.05 was considered statistically significant.

## Results

**Fig. 1:**
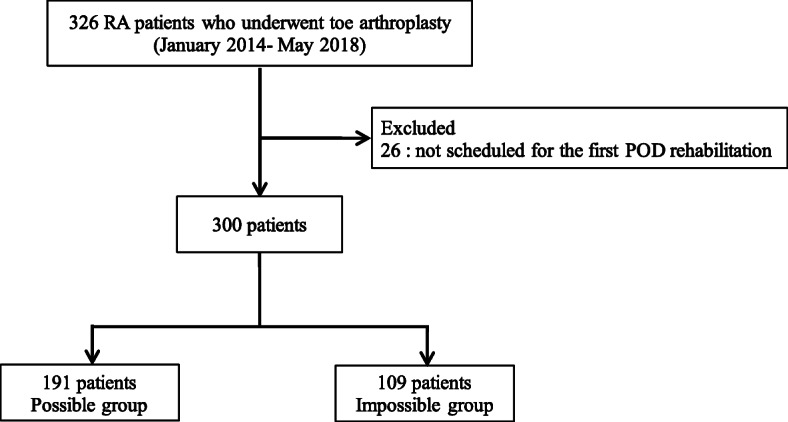
Flow chart of patients selection. RA, rheumatoid arthritis; POD, postoperative day

During the study period, 326 patients were reviewed and 300 patients were included in the analysis: possible group (*n* = 191) and impossible group (*n* = 109) (Fig. 1). Overall, 36.3% of patients were unable to walk during rehabilitation on the first POD.

The patients’ demographic and perioperative data are shown in Table 1. No significant differences were observed in age, sex, height, or body weight between the two groups. The number of preoperative anti-rheumatic drugs, prednisolone and DMARDs, was similar between the two groups. Surgeries were performed by only one surgical team in the two groups. The proportion managed with PNB was higher, and the incidence of PONV and number of rescue analgesics administered before rehabilitation were lower in the possible group than in the impossible group. Almost all rescue analgesics were either intravenous flubiprofen or diclofenac suppository.

**Table 1:** Patients’ demographic and perioperative data

Variables	Possible group	Impossible group	*P* value
*N*	191	109	
Age (years)	63.7 (10.6)	62.1 (10.1)	0.20
Male/female	14/177	4/105	0.20
Height (cm)	155.2 (7.2)	156.1 (7.2)	0.30
Weight (kg)	51.3 (9.0)	51.3 (8.6)	0.99
Medications			
Daily prednisolone medication, *n* (%)	81 (42.4%)	36 (33.0)	0.11
Daily prednisolone dose (mg)	0 [0–2.75]	0 [0–2.25]	0.22
Number of DMARDs taken by patients	1 [1–2]	1 [1–2]	0.37
Duration of surgery (min)	102.2 (37.4)	109.6 (34.1)	0.09
Number of operated toes			
Duration of anesthesia (min)	154.4 (42.3)	162.3 (39.5)	0.11
Intraoperative fluid volume (ml)	918.4 (306.6)	1000.5 (329.5)	0.03
Blood loss (g)	2.3 (5.3)	3.9 (8.0)	0.03
Anesthesia methods			
General anesthesia, *n* (%)	160 (83.8%)	89 (81.7%)	0.64
Regional anesthesia, n (%)	105 (55.0%)	47 (43.1%)	0.05
Epidural anesthesia, *n* (%)	28 (14.7%)	21 (19.3%)	0.30
Spinal anesthesia, *n* (%)	31 (16.3%)	20 (18.3%)	0.64
Peripheral nerve block, *n* (%)	58 (30.1%)	13 (11.9%)	< 0.001
Intraoperative fentanyl dose (μg)	250 [0–400]	300 [50–400]	0.14
Postoperative fentanyl use, *n* (%)	75 (39.3%)	57 (52.3%)	0.03
Incidence of PONV before rehabilitation, *n* (%)	42 (22.0%)	47 (43.1%)	< 0.001
Number of rescue analgesics administered before rehabilitation	1 [0–1]	1 [0–2]	0.01
Days from surgery to discharge	14.7 (0.4)	14.7 (0.6)	0.94

Data are presented as mean (SD), median [interquartile range], or number (%)

*DMARDs* disease-modifying anti-rheumatic drugs, *PONV* postoperative nausea and vomiting

The primary outcomes are shown in Table 2. The incidence of PONV before rehabilitation was significantly associated with the infeasibility of walking rehabilitation on the first POD (OR 2.43; 95% CI 1.44–4.14; *P* = 0.003). The number of rescue analgesics administered before rehabilitation was associated with the infeasibility of walking rehabilitation on the first POD (OR 1.29; 95% CI 1.04–1.59; *P* = 0.018). PNB was also associated with the infeasibility of walking rehabilitation on the first POD (OR 0.41; 95% CI 0.20–0.79; *P* = 0.010). Age, sex, daily prednisolone use, intraoperative fentanyl dose, and postoperative fentanyl use were candidate variables, but were not included in the model. The correlation coefficient between postoperative fentanyl use and PONV was 0.4 and was not utilized.

**Table 2:** Primary and secondary outcomes

Primary outcomes Logistic regression analysis for infeasibility of walking rehabilitation on the first POD			
Variables	**Adjusted odds ratio**	**95% CI**	***P*value**
Incidence of PONV before rehabilitation	2.43	1.44–4.14	0.003*
Peripheral nerve block	0.41	0.20–0.79	0.010*
Number of rescue analgesics administered before rehabilitation	1.29	1.04–1.59	0.018*
Variables	**Non-adjusted odds ratio**	**95% CI**	***P*value**
Incidence of PONV before rehabilitation	2.71	1.63–4.50	< 0.001
Peripheral nerve block	0.31	0.16–0.60	< 0.001
Number of rescue analgesics administered before rehabilitation	1.35	1.11–1.66	0.003
Age (years)	0.99	0.96–1.01	0.20
male	0.48	0.15–1.50	0.18
Daily prednisolone medication	0.58	0.30–1.11	0.10
Epidural anesthesia	1.39	0.75–2.59	0.30
Intraoperative fentanyl dose (μg)	1.00	0.99–1.00	0.13
Postoperative fentanyl use	1.70	1.05–2.73	0.03
Secondary outcomes			
Variables	**Possible group**	**Impossible group**	***P*value**
*N*	191	109	
Time to first rescue analgesic requirement (min), median [IQR]	1320 [528–no use]	1046 [366–1897]	0.03
Number of rescue analgesics administered during the first 3 PODs median [IQR]	1 [0–3]	3 [1–5]	0.004

Secondary outcomes are presented as median [interquartile range]

*POD* postoperative day, *CI* confidence interval, *IQR* interquartile range, *PONV* postoperative nausea and vomiting

*Calculated with the Benjamini–Hochberg method

**Fig. 2:**
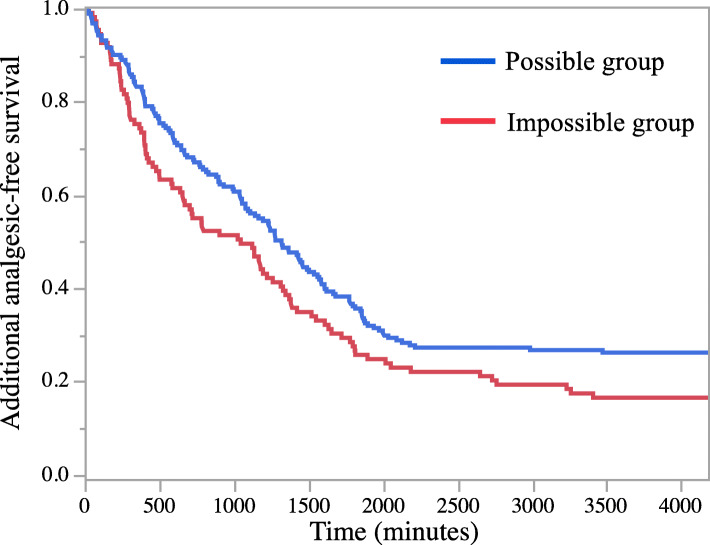
Kaplan–Meier curves for analgesic-free survival after surgery for both groups

The secondary outcomes are shown in Table 2. A survival curve of the first pain rescue after the surgery is shown in Fig. 2. The median survival time to the first pain rescue was longer in the possible group compared to the impossible group (1320 min vs. 1046 min, *P* = 0.03). The number of rescue analgesics during the first 3 PODs was lower in the possible group than in the impossible group (1 [0–3] vs. 3 [1–5], *P* = 0.004). The post hoc analysis is shown in Table 3. Among the four anesthesia methods (GA, GA + IVPCA with fentanyl, GA + epidural anesthesia, GA + PNB), GA + PNB had the lowest ratio of PONV (11.3%, *P* < 0.0001) and the infeasibility of walking rehabilitation (17.7%, *P* = 0.0029).

**Table 3:** Post hoc analysis between anesthesia methods and postoperative outcome

	Anesthesia methods				*P* value
	GA	GA + IVPCA	GA + Epi	GA + PNB	
*N*	33	115	30	62	
Infeasibility of walking rehabilitation on the first POD	11 (33.3%)	51 (44.3%)	14 (46.7%)	11 (17.7%)	0.0029
Incidence of PONV before rehabilitation, *n* (%)	7 (21.2%)	56 (48.7%)	8 (26.7%)	7 (11.3%)	< 0.0001
Number of rescue analgesics administered during the first 3 PODs	1 [1–4]	1 [0–4]	1 [0–3.25]	2[0–3]	0.32

Data are presented as median [interquartile range] or number (%)

*GA* general anesthesia, *IVPCA* intravenous patient-controlled analgesia, *Epi* epidural anesthesia, *PNB* peripheral nerve block, *POD* postoperative day, *PONV* postoperative nausea and vomiting

## Discussion

The present study showed that, in RA patients who underwent toe arthroplasty, the incidence of PONV and inadequate postoperative pain management were predictors for the infeasibility of walking rehabilitation on the first POD, which was consistent with part of our hypothesis. PNB was a preventive factor for the infeasibility of walking rehabilitation on the first POD. In contrast, the use of daily oral steroids and number of DMARDs, which may be associated with activities of daily living and the severity of RA, were not related to postoperative rehabilitation. The entire study population suffered from severe RA with a positive surgical intervention. In addition, the study population postoperatively maintained general functional ability. As our institute is one of the national large-scale rheumatoid centers, anti-rheumatoid medication therapy was well optimized, and there was a relatively small difference in the severity of disease among the patients.

Early postoperative rehabilitation was affected by PONV [6]. The incidence of PONV varied from 20 to 80% [7, 8] and was affected by surgical time, sex, and postoperative opioids [9]. Since a large proportion of rheumatoid patients were women, they were expected to be at a high risk for PONV. In our study population, the ratio of women was 96%, and among them, 29.7% developed PONV. Although IVPCA with fentanyl would provide good management of postoperative pain, continuous postoperative opioid use was a risk factor of PONV [9]. In post hoc analysis, continuous postoperative intravenous fentanyl doubled the incidence of PONV; the choice of regional anesthesia rather than IVPCA for postoperative pain management helped prevent the incident of PONV.

In our study, multivariate analysis showed an association between early rehabilitation and postoperative pain, and this was consistent with a previous report in total knee arthroplasty cases of non-RA patients [10]. Generally, postoperative pain control is evaluated using pain scores, such as the visualized analogue scale and numerical rating scale. However, the number of pain rescue medications was selected as a factor in our logistic regression model. Due to the retrospective design of our study, the timing of pain evaluation may be inconsistent, and we considered the number of pain rescue medications to be a more precise and objective measure. Our Kaplan–Meier curves also showed that the time of the first analgesic rescue was associated with early rehabilitation. A longer duration of postoperative analgesia and multimodal analgesia is the key to early rehabilitation.

The present study has several limitations. It was a retrospective, single-center study, and patients were not randomized. Choice of anesthesia modality was determined by the anesthesiologists in charge, according to the experience of the anesthesiologist. As our institute is an educational institute, regional anesthesia technique might not be performed consistently. The PNB included several types and dosages of local anesthetics. Surgery without general anesthesia was not common in our hospital and could not be compared to other modalities in this study, but such anesthesia management may be effective. It was guessed that avoidance of inhalation anesthesia and intraoperative uses of fentanyl dosage were also important factors for improving rehabilitation. However, this study could not investigate these factors because the sample size was not sufficient to determine the details of general anesthesia as a factor. We did not investigate the severity of RA. Instead, we evaluated the grade of severity of RA by medicines taken by the patients. Further prospective studies are required to further evaluate the relationship between anesthesia modality and early rehabilitation in RA patients.

In summary, anesthesia method strongly affects postoperative rehabilitation and postoperative pain management. Continuous epidural anesthesia sometimes results in bilateral motor block, which disturbs early mobilization after surgery and limits early rehabilitation. The choice of PNB instead of IVPCA or epidural anesthesia may have an advantage in early rehabilitation.

## Conclusion

The incidence of PONV and inadequate postoperative pain management were predictors for the infeasibility of walking rehabilitation on the first POD. For postoperative rehabilitation after rheumatoid toe surgery, anesthesia method to prevent PONV and providing good postoperative pain management are important. In addition, adding PNB to GA would decrease PONV and improve postoperative pain management.

## Data Availability

The datasets used and/or analyzed during the current study are available from the corresponding author upon reasonable request.

## Abbreviations

CI: Confidence interval
DMARDs: Disease modifying anti-rheumatic drugs
GA: General anesthesia
IVPCA: intravenous patient controlled analgesia
OR: Odds ratio
PNB: Peripheral nerve block
POD: Postoperative day
PONV: Postoperative nausea and vomiting
RA: Rheumatoid arthritis
